# Through the Parent’s Eyes: Exploring the Relationship Between Parental Perceptions of Difficulties and SDQ Scale Results in Children and Adolescents

**DOI:** 10.1192/j.eurpsy.2024.737

**Published:** 2024-08-27

**Authors:** A. S. Gonçalves, R. Rodrigues, F. Monteleone, B. Couto, R. Ortiga, V. Rocha

**Affiliations:** ^1^Service of Psychiatry; ^2^Service of Child and Adolescent Psychiatry, Hospital da Senhora da Oliveira Guimarães, Guimarães, Portugal

## Abstract

**Introduction:**

The Strengths and Difficulties Questionnaire (SDQ) is a widely used assessment tool for measuring the psychological well-being of children and adolescents. It consists of 25 items that assess emotional symptoms, conduct problems, hyperactivity/inattention, peer relationship difficulties, and prosocial behavior.

**Objectives:**

The present study aimed to investigate the relationship between parental perceptions of difficulties and the results obtained from the SDQ.

**Methods:**

Participants were recruited from the initial consultation of Child and Adolescent Psychiatry (N=132). Parents completed a questionnaire assessing their subjective perceptions of their child’s difficulties in various domains (home, school, learning activities, relations with friends) on a 4-point scale ranging from “no notion of difficulties” to “very severe difficulties”. They also completed the SDQ scale. Data were analyzed using SPSS software.

**Results:**

In this study, 74% of participants had scores on the SDQ indicating potential psychological difficulties. Additionally, 17.4% of participants had scores on the borderline between normal and abnormal results. 47% of patients scored above the cut-line for problems on the hyperactivity/inattention subscale, indicating higher levels of difficulties in this area. Conversely, only 3% of participants scored problematic scores on the peer relationship difficulties subscale. There was no statistical difference between sexes in terms of SDQ scores. A correlation analysis revealed a significant positive correlation (p < 0.01) between parental perceptions of difficulties and higher SDQ scores and the mean score on the SDQ scale was found to be significantly higher in patients who were rescheduled for another consultation following the evaluation by doctors, compared to those patients who received clinical discharge from the initial consultation (p-value 0,040).

**Conclusions:**

This study provides valuable insights into the concordance between parental perceptions and objective assessments of difficulties in children and adolescents. Parents who perceived their child to have more difficulties also reported higher levels of psychological difficulties on the SDQ. This study highlights the importance of using tools like the SDQ to assess psychological well-being in children and adolescents. It also emphasizes the practical utility of the SDQ as a time-efficient assessment tool for use during initial consultations in child and adolescent psychiatry.

**Disclosure of Interest:**

None Declared

